# Molecular dynamic simulations to investigate the structural impact of known drug resistance mutations on HIV-1C Integrase-Dolutegravir binding

**DOI:** 10.1371/journal.pone.0223464

**Published:** 2020-05-07

**Authors:** Rumbidzai Chitongo, Adetayo Emmanuel Obasa, Sello Given Mikasi, Graeme Brendon Jacobs, Ruben Cloete

**Affiliations:** 1 South African Medical Research Council Bioinformatics Unit, South African National Bioinformatics Institute, University of the Western Cape, Cape Town, South Africa; 2 Division of Medical Virology, Department of Pathology, Faculty of Medicine and Health Sciences, Stellenbosch University, Tygerberg, Cape Town, South Africa; Bioinformatics Institute, SINGAPORE

## Abstract

Resistance associated mutations (RAMs) threaten the long-term success of combination antiretroviral therapy (cART) outcomes for HIV-1 treatment. HIV-1 Integrase (IN) strand transfer inhibitors (INSTIs) have proven to be a viable option for highly specific HIV-1 therapy. The INSTI, Dolutegravir is recommended by the World Health Organization for use as first-line cART. This study aims to understand how RAMs affect the stability of IN, as well as the binding of the drug Dolutegravir to the catalytic pocket of the protein. A homology model of HIV-1 subtype C IN was successfully constructed and validated. The site directed mutator webserver was used to predict destabilizing and/or stabilizing effects of known RAMs while FoldX confirmed any changes in protein energy upon introduction of mutation. Also, interaction analysis was performed between neighbouring residues. Three mutations known to be associated with Raltegravir, Elvitegravir and Dolutegravir resistance were selected; E92Q, G140S and Y143R, for molecular dynamics simulations. The structural quality assessment indicated high reliability of the HIV-1C IN tetrameric structure, with more than 90% confidence in modelled regions. Change in free energy for the three mutants indicated different effects, while simulation analysis showed G140S to have the largest affect on protein stability and flexibility. This was further supported by weaker non-bonded pairwise interaction energy and binding free energy values between the drug DTG and E92Q, Y143R and G140S mutants suggesting reduced binding affinity, as indicated by interaction analysis in comparison to the WT. Our findings suggest the G140S mutant has the strongest effect on the HIV-1C IN protein structure and Dolutegravir binding. To the best of our knowledge, this is the first study that uses the consensus wild type HIV-1C IN sequence to build an accurate 3D model to understand the effect of three known mutations on DTG drug binding in a South Africa context.

## Introduction

The Integrase (IN) enzyme plays an important role in the Human Immunodeficiency Virus type 1 (HIV-1) replication cycle by catalysing two distinct reactions termed: 3’-end processing and strand transfer. During the 3’ processing, IN removes two nucleotides from the 3’ ends of both viral DNA strands and exposes the C-alpha hydroxyl group on the 3’ends. The subsequent step involves strand transfer whereby, IN attacks the phosphodiester backbone of the host DNA and links the exposed 3’-end to the 5’ hydroxyl end of the host DNA [1]. This makes HIV-1 IN an important target for combination antiretroviral therapy (cART). HIV-1 IN is a 32 kilo Dalton (kDa) protein, and consist of three structural and functional domains; the N-terminal domain (NTD, residues 1–49), the catalytic core domain (CCD, residues 50–212), and C-terminal domain (CTD, residues 213–288). It also contains a conserved DDE motif consisting of residues Asp64, Asp116 and Glu152 in the CCD, important for drug binding and enzyme activity [2]. Several IN strand transfer inhibitors (INSTIs) have been developed [3–5]. These inhibitors include; Raltegravir (RAL) and Elvitegravir (EVG) as first-generation INSTIs and Dolutegravir (DTG) and Bictegravir (BIC) are second-generation inhibitors [6]. All first-generation INSTIs have been reported to have relatively low genetic barrier to resistance while second-generation INSTIs including DTG (a coplanar and linear molecule) are associated with a higher genetic barrier against resistance associated mutations (RAMs), and are rendered safe and tolerable, showing little to none drug-drug interactions resistance [7].

The functional mechanism of INSTIs is to bind to the catalytically essential magnesium ions, thereby displacing the reactive 3’-hydroxyl group of the terminal A17 away from the active site which disrupts the strand transfer process. Several mutations have emerged in patients receiving first-line INSTIs, RAL and EVG. Brado *et al*. reported that despite higher fold RAMs against INSTIs being absent in most treatment naïve patients, they can emerge under treatment, particularly with first generation INSTIs [8].

Genetic resistance pathways including primary mutations at codons Y143C/H/R, Q148H/K/R or N155H together with one or more additional associated secondary mutations at L74M, E92Q, T97A, E138E/A/K or G140S/A, has been reported to result in higher levels of resistance with RAL treatment [9–11]. On its own, the Y143R non-polymorphic mutation reduces RAL susceptibility by ~20-fold, but has no effect on DTG susceptibility [12,13]. On the other hand, EVG specific resistance pathways involve IN mutations T66I/A/K, E92Q/G and S147G pathways [14,15]. The E92Q mutation alone reduces EVG susceptibility to >20-fold and results in limited (<5-fold) cross-resistance to RAL [14–16]. The E92Q mutation is also selected *in vitro* by DTG and reduces DTG susceptibility by ~1.5-fold [11, 17]. With more people living with HIV-1 in resource limited countries still receiving RAL and EVG treatment as first-line anti-retroviral ARV therapy, these treatments have suffered an extensive cross-resistance of mutations, highlighting the need for a switch to INSTIs possessing a more robust resistance profile such as DTG. Furthermore, a recent study provided evidence for the replacement of RAL with DTG based on the low prevalence of DTG resistance and the low risk for INSTI mutations when patients are on DTG treatment [18]. Several studies have used the prototype foamy virus intasome structure (medium sequence identity) to model the structure of HIV-1 IN in order to investigate the effect of single and double mutations on HIV-1 IN and drug binding using molecular dynamic simulations [19–23]. Here, molecular dynamics studies have demonstrated the importance of this 140’s loop’s flexibility as a mechanism of drug resistance [19–23]. However, the findings from some studies were inconclusive due to the poor quality of the protein models delineating the active site and viral DNA binding site for simulation studies.

HIV-1 subtype C (HIV-1C) accounts for nearly 50% of all global HIV-1 infections, while HIV-1 subtype B (HIV-1B) accounts for only approximately 12% [24]. However, a vast majority of research on HIV-1 infections focussed on the development of combination anti-retroviral therapy (cART) drugs for HIV-1B and studying the mechanisms of drug resistance in HIV-1B, with less information known about HIV-1C. As a result, all antiretroviral drugs have been developed in relation to HIV-1B. They have also been reported to be effective against a wide range of HIV-1 subtypes [25]. Other clinical studies have however revealed very poor cART treatment outcome when associated with HIV-1C infections [26–28]. Although HIV-1C has not been considered an effective predictor for therapy failure earlier, a recent trial indicated that HIV-1C has independent predictors for viral failure [28]. Recent studies also have identified subtype specific differences in DTG cross-resistance pattern in patients failing the first-generation RAL treatment [8,14].

Our work carries on from such previously reported molecular dynamics simulation findings to try and assess the molecular mechanisms of resistance in a subtype C IN protein and see how the reported known resistance mutations will affect binding affinity. Based on this insight, this study has been dedicated to employ *in silico* methods to understand whether the second-generation drug, DTG, will be able to retain efficacy against selected RAL and EVG known resistance mutations in an HIV-1C IN protein. In 2017, Cryogenic electron microscopy was used to solve the structure of HIV-1 strand transfer complex intasome for HIV-1 subtype B [29]. This provided us with a unique opportunity to model the structure of HIV-1 subtype C IN to interrogate the effect of known drug resistance associated mutations (RAMs) on the protein structure using molecular dynamic simulation studies. This is the first study that uses the consensus wild type subtype C IN sequence to build an accurate 3D model of HIV-1C IN to understand the effect of three known RAL, EVG and DTG mutations on DTG drug binding.

## Materials and methods

### Generation of consensus HIV-1C Integrase sequence

To compare our sequences with the rest of the IN sequences from South Africa, we performed a search on the HIV Los Alamos National Library (LANL) database (https://www.hiv.lanl.gov/components/sequence/HIVsearch.com). Our search inclusion criteria included all South African HIV-1 subtype C IN sequences and those identified from treatment naïve patients. We selected one sequence per patient and all problematic sequences were excluded from further analyses. Finally, the consensus sequence was generated using the database-derived HIV-1C_ZA_ sequences (**n = 314**) and cohort sequences (**n = 91**) [8]. Nucleotide sequences were verified for stop codons, insertion and deletions using an online quality control program on the HIVLANL database (https://www.hiv.lanl.gov/content/sequence/QC/index.htm). Multiple sequence alignments were done with MAFFT version 7, from which the consensus sequence was derived [30]. As part of quality control, each of the viral sequences were inferred on a phylogenetic tree in order to eliminate possible contamination.

### Molecular modelling and quality assessment

The crystal structure of the HIV-1B intasome (PDBID: 5U1C) was used to generate a three-dimensional tetrameric structure of HIV-1C IN using the consensus HIV-1C sequence that we generated. The SWISSMODEL webserver was used for model generation by first constructing a pairwise sequence-structure alignment between HIV-1C wild-type (WT) amino acid sequence and template 5U1C [31]. The quality of the resulting model was assessed using SWISSMODEL quality assessment scores, Root mean square deviation analysis compared to homologous template (PDBID: 5U1C) and with publicly available algorithms located at the SAVES webserver (https://servicesn.mbi.ucla.edu/SAVES/) namely; ERRAT, VERIFY3D and Ramachandran plot [32–34].

### Structure preparation

The predicted 3D structure of HIV-1C IN was superimposed to 5U1C to extract proviral DNA, while the Magnesium (MG) ions and drug DTG were extracted and obtained from homologous template 3S3M (Prototype foamy virus) onto their specific positions within the predicted HIV-1C IN using PyMOL. The atomic coordinates of the wild-type (WT) structure of HIV-1C in complex with DNA, MG and DTG, were uploaded onto the CHARMM-GUI solution builder webserver to generate a series of input files for energy minimization of the molecule in an aqueous solvent environment [35, 36]. 50,000 steps of energy minimization using the steepest descent minimization integrator was used to equilibrate the system of the solvated complex structure using CHARMM36 force field [37], and applying constraints to hydrogen bonds using the LINCS constraint algorithm. All this was performed with Gromacs software version 5.1 [38]. Thereafter, we predicted the stabilizing and/or destabilizing effect of mutations on the protein structure. For this purpose, the site directed mutator (SDM) webserver and the software FoldX was used to predict the change in Gibbs free energy after the introduction of the mutation [39, 40]. We also calculated the loss or gain of polar interactions between neighbouring residues located adjacent to the mutation using PyMOL [36].

### Molecular dynamic simulation

For simulation studies we only considered the two inner dimers of the protein structure, as the other two monomers were similar in sequence and structure. Three different mutant systems were prepared by introducing a specific mutation into the WT structure through the mutagenesis wizard in PyMOL and energy minimizing the structures using Gromacs applying the same parameters used to energy minimize the WT structure [38, 41]. The WT and three mutant systems (E92Q, G140S and Y143R) were prepared by uploading the atomic coordinates of the Protein-DNA-MG-DTG complexes to the CHARMM-GUI interface [36]. These three mutant systems were selected for simulation studies because they represent three resistance pathways associated with RAL, EVG and possibly DTG resistance. Both E92Q and G140S mutations have been reported to reduce susceptibility of DTG ~1.5-fold and up to 10-fold respectively [13,17]. The individual systems were built using the solution builder option in the input generator [35,36]. Each system was solvated in a rectangular TIP3 water-box with 10Å distance between the edges of the box. The topology and coordinates for each system was generated using the CHARMM36 all-atom force field [37] and CHARMM general force field [37] for DTG. Each system was neutralized by adding counter ions to each of the systems. For the WT system, 157 potassium ions (K) and 81 chloride ions (Cl) were added, for the mutant Y143R system we added 156 K and 81 Cl ions, while for the mutant system G140S we added 157 K and 81 Cl ions and for the mutant system E92Q we added 156 K and 81 Cl ions. Each system was at a final concentration of 0.15M for simulation dynamics.

Gromacs version 5.1 was used for running all the simulations [38]. Each system underwent 50000 steps of steepest descents energy minimization to remove steric overlap. Afterwards, all the systems were subjected to a two-step equilibration phase namely NVT (constant number of particles, Volume and Temperature) and NPT (constant Number of particles, Pressure and Temperature). The NVT equilibration was run for 500 picoseconds (ps) to stabilize the temperature of the system and a short position restraint NPT was run for 500 ps to stabilize the pressure of the system by relaxing the system and keeping the protein restrained. The V-rescale temperature-coupling [38,39] method was used for the NVT ensemble, with constant coupling of 1 ps at 303.15K. For NPT, the Nose-Hoover pressure coupling [42–44] was turned on with constant coupling of 1ps at 303.15K under conditions of position restraints (h-bonds) selecting a random seed. Electrostatic forces were calculated for both NVT and NPT using Particle Mesh Ewald method [45]. All systems were subjected to a full 300 nanoseconds (ns) simulation under conditions of no restraints, an integration time step of 0.002 ps and an xtc collection interval of 5000 steps for 10 ps. Each simulation was repeated (duplicating each simulation separately) to validate reproducibility of results.

The analyses of the trajectory files were done using GROMACS utilities. The root mean square deviation (RMSD) was calculated using **gmx rmsd** and root mean square fluctuation (RMSF) analysis using **gmx rmsf**. The radius of gyration was calculated using **gmx gyrate** to determine if the system reached convergence over the 300 ns simulation. Pairwise distance analysis between the drug and MG was done using **gmx pairdist** tool. The total number of hydrogen bonds between the protein and drug was calculated using **gmx hbond**. The total pairwise non-bonded interaction energy (which is not a free energy or binding energy) between the protein and the drug DTG was calculated using gmx energy over 100 ns. The energy terms included were average short range Coulombic interactions and short range Lennard Jones interactions. The free energy of binding was calculated using Molecular mechanics Poisson–Boltzmann surface area (MM-PBSA) protocols implemented in the g_mmpbsa package [46]. The ΔG binding energy was calculated for 800 frames between the protein and the drug over 8 ns from 100–108 ns of the simulation trajectory with a sampling interval of 10 ps. Afterwards, we extracted structures every 50 ns over the last 200 ns of the equilibrated system to determine any structural changes and differences in the number of interactions between the protein and drug at different time intervals.

### Principal component analysis

Principal component analysis (PCA) is a statistical technique that reduces the complexity of a data set in order to extract biologically relevant movements of protein domains from irrelevant localized motions of atoms. The technique is known for its ability to transform a number of (possibly) correlated variables into a (smaller) number of uncorrelated variables, called principal components (PCs), while retaining those characteristics of the data set that contribute most to its variance [19]. For PCA analysis the translational and rotational movements were removed from the system using **gmx covar** from GROMACS to construct a covariance matrix. Next the eigenvectors and eigenvalues were calculated by diagonalizing the matrix. The eigenvectors that correspond to the largest eigenvalues are called "principal components", as they represent the largest-amplitude collective motions. We filtered the original trajectory and project out the part along the most important eigenvectors namely: vector 1 and 2 using **gmx anaeig** from GROMACS utilities. Furthermore, we visualized the sampled conformations in the subspace along the first two eigenvectors using **gmx anaeig** in a two-dimensional projection.

## Results

### Sequence and structural analysis

**Fig 1:**
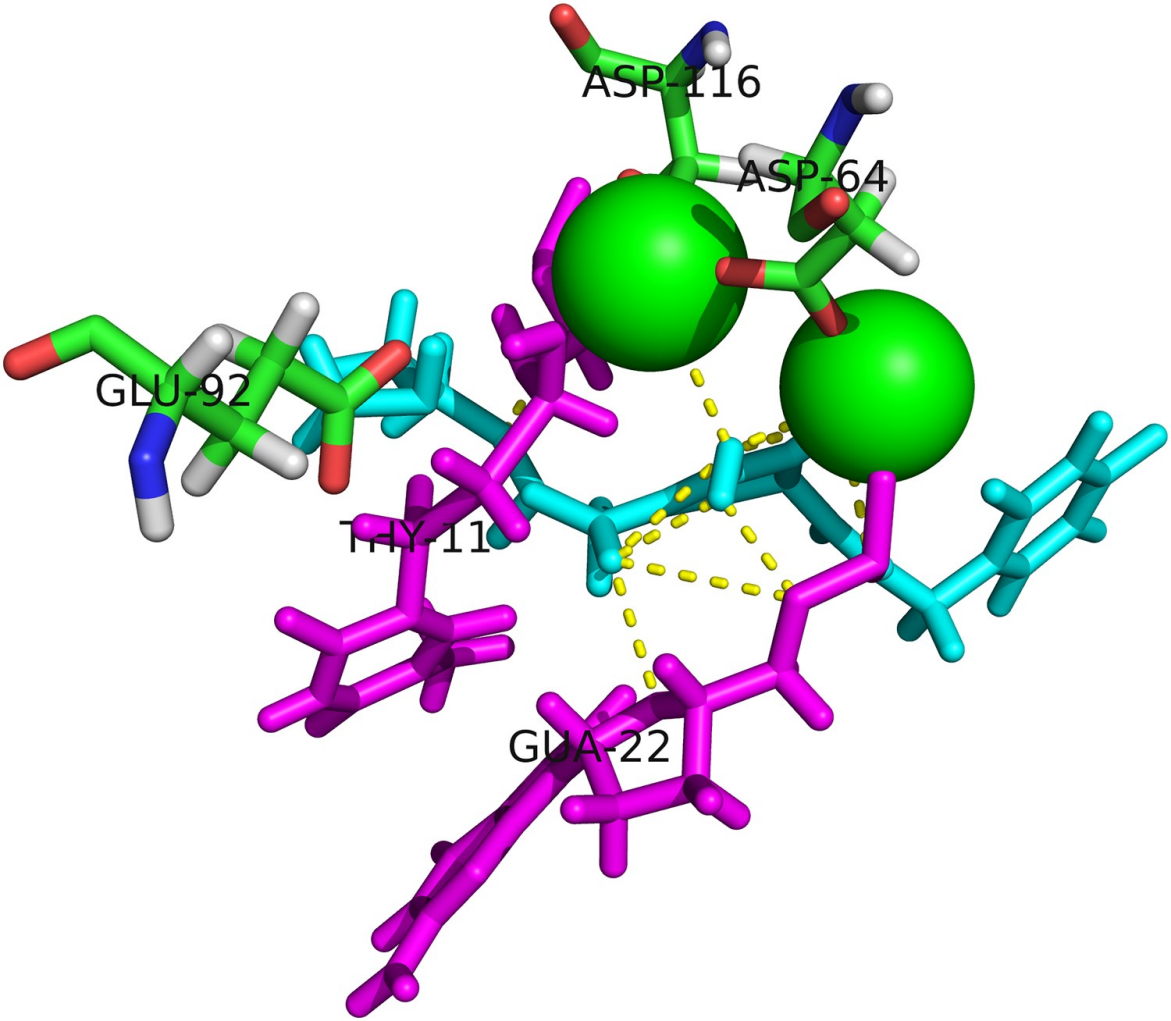
HIV-1C Integrase active site showing interactions with DNA, MG and drug Dolutegravir. Magnesium^2+^ ions (green spheres) are shown sitting in close proximity with Dolutegravir (cyan) within the binding pocket (DDE motif) residues are labelled and shown as sticks. Two DNA residues: THY11 and GUA22 (magenta sticks) are shown expressing polar interactions with Dolutegravir and the drug also interacts with both MG ions as shown. Dashed yellow lines show polar contacts.

The amino acid sequence of HIV-1C IN shared 93.4% sequence identity with the template 5U1C amino acid sequence (S1 Fig). The tetrameric protein structure built for HIV-1C IN had a Global mean quality estimate score of 0.59 and 60% sequence similarity to 5U1C (S2 Fig). The active site residues, MG and DNA nucleotides involved in DTG binding to HIV-1C IN are shown in Fig 1. The VERIFY 3D score was 80.1%, the ERRAT overall quality score was 90% and higher for all four chains (A, B, C and D) and the Ramachandran plot indicated more than 90% of residues fell within the most favoured regions of the plot suggesting the predicted structure is a reliable model. Stability predictions indicated 15 RAMs to be destabilizing and five to be stabilizing for the protein structure based on SDM delta-delta G free energy scores (Table 1). The FoldX change in unfolded energy values indicated that the G140S was stabilizing, E92Q destabilizing and Y143R neutral based on comparison with the WT structure each having values of 162.89, 131.94, 146.47 and 151.83 Kcal/Mol, respectively. Interaction analysis showed ten mutations resulted in a loss of polar contacts; three resulted in an increase in polar contacts, while seven showed no change in the number of polar contacts with neighbouring residues (Table 1).

**Table 1:** Summary of stability predictions and polar interactions.

	SDM^1^	FoldX^2^		Polar Interactions	
Mutation	Predicted ΔG (Kcal/Mol)	Total Energy ΔG (Kcal/Mol)	Energy Difference	Wild Type	Mutant
WT	N.A	151.83	N.A	N.A	N.A
T66A	-1.2	152.69	0.86	3 (H67, I73, ADE21)	1 (I73)
T66I	0.08	153.63	1.80	3 (H67, I73, ADE21)	1 (I73)
T66K	-0.61	160.15	8.32	3 (H67, I73, ADE21)	1 (I73)
E92Q	-0.16	131.94	-19.89	3 (Q136, I113, T115)	0
E138K	-0.12	151.32	-0.51	3 (Q136, I113, T115)	2 (T115, I113)
E138A	-0.4	152.01	0.18	3 (Q136, I113, T115)	2 (T115, I113)
E138T	0.48	151.82	-0.01	3 (Q136, I113, T115)	2 (T115, I113)
G140S	-0.58	162.89	11.06	2 (T115, N117)	3 (Q148, T115, N117)
G140A	-0.68	152.43	0.60	2 (T115, N117)	2(T115, N117)
G140C	0.39	154.81	2.98	2 (T115, N117)	2(T115, N117)
Y143C	0.14	152.49	0.66	None	None
Y143R	-0.08	146.47	-5.36	None	1 (S230)
Y143H	-0.07	152.28	0.45	None	None
S147G	-0.18	151.16	-0.67	2 (Q148, N144)	1 (Q144)
Q148H	0.63	157.78	5.95	3 (V151, P145, S147)	2 (V151, P145)
Q148K	-0.78	151.33	-0.50	3 (V151, P145, S147)	3 (V151, P145, H114)
Q148R	-0.71	152.53	0.70	3 (V151, P145, S147)	4 (D116,P145, V150, V151)
Q148N	-0.82	151.58	-0.25	3 (V151, P145, S147)	3 (V151, P145, H114)
N155H	-0.23	152.01	0.18	3 (V151, P145, S147)	3 (E152, V151, K159)
R263K	-0.29	151.15	-0.68	4 (Q146, N144, GUA18, ADE18)	0

^1^negative values for ΔΔG indicate a stabilizing effect and positive values destabilizing.

^2^positive energy difference ΔG values >1 indicate a destabilizing effect, whereas values 1 ≤ ΔG ≤ 0 imply a neutral effect and ΔG values > -1 indicate a stabilizing effect. Abbreviations used: N.A- not applicable. The number in front of brackets is the total amount of interactions. Abbreviations of amino acids: A -Alanine; D-Aspartic acid; E-Glutamic acid; G-Glycine; H-Histidine; I-Isoleucine; K-Lysine; N-Asparagine; Q-Glutamine; R-Arginine; S-Serine; T-Threonine; Y-Tyrosine.

### Molecular dynamic simulations

**Fig 2:**
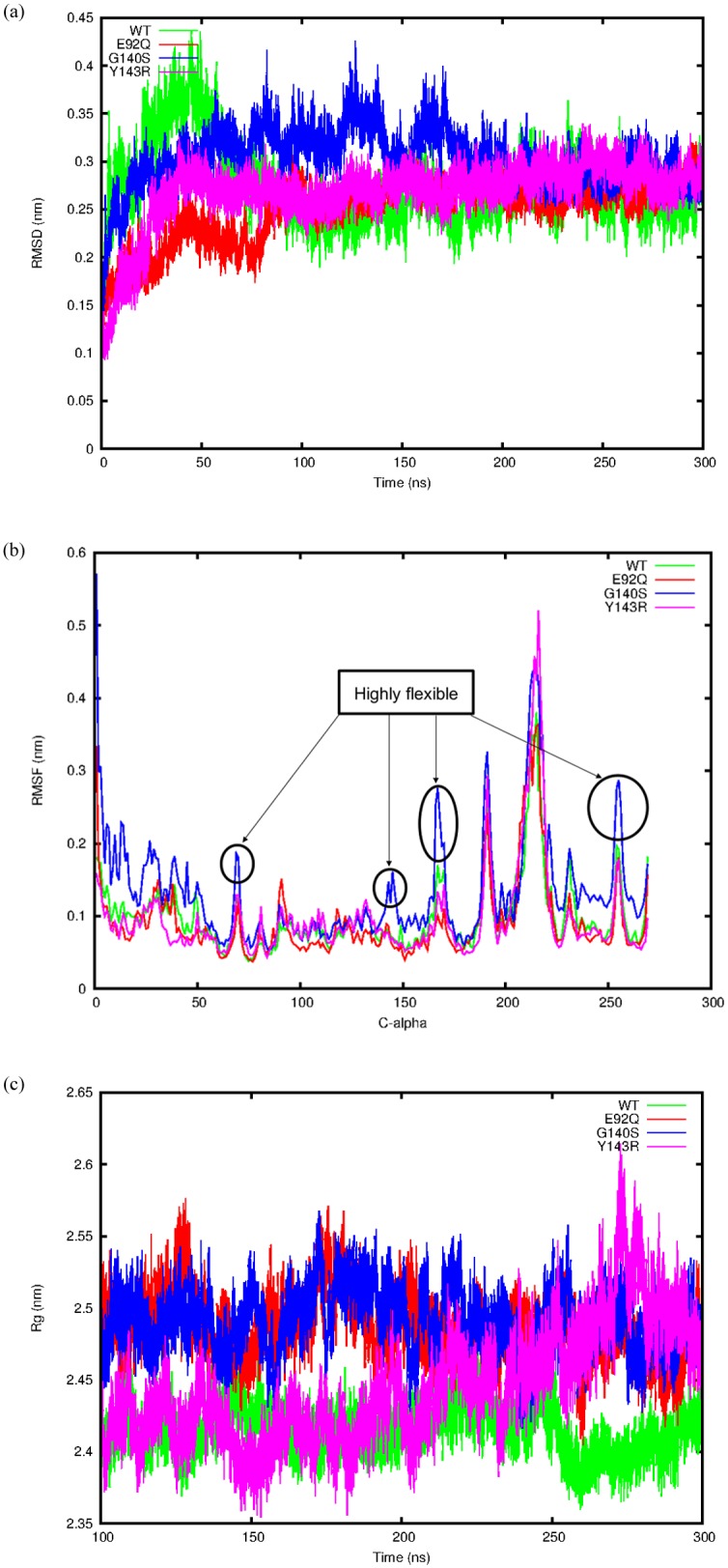
Trajectory analysis of the four simulation systems. (A) Change in backbone RMSD for the WT, E92Q, G140S and Y143R systems plotted over 300 ns. (B) Change in RMSF for the C-alpha residues for the WT, E92Q, G140S and Y143R systems plotted over the last 200ns. (C) Measure of compactness for the WT, E92Q, G140S and Y143R systems plotted over the last 200 ns.

All the MD trajectory analysis considered the single chain A (monomer) of the IN protein in contact with the drug DTG and the DNA. Trajectory analysis of the RMSD of the backbone indicated that the WT system reached equilibrium after 100 ns as well as the E92Q, Y143R and G140S mutant systems (Fig 2A). Only G140S showed higher RMSD variation values compared to the WT, Y143R and E92Q systems (Fig 2A). RMSF analysis clearly showed higher flexibility for the G140S mutant system, with four highly flexible regions (residues 68–70, 142–146, 166–170 and 253–256) compared to the WT, E92Q and Y143R systems (Fig 2B). These flexible regions affect the 140’s loop region that regulates drug binding. The Radius of gyration values indicated decreasing values for Y143R and E92Q compared to the WT and G140S mutant system (Fig 2C). Plotting the first two principal components provided insight into the collective movement of each protein atom. The 2D projections of the first and second principal components for the WT vs E92Q, WT vs Y143R and WT vs G140S systems are shown in S3A, S3B and S3C Fig. Calculation of the covariance matrix values after diagonalization showed a significant increase for the G140S system (18.33 nm) compared to the other three systems WT, E92Q and Y143R each having 9.58 nm, 8.98 nm and 10.41 nm lower values, respectively. Distance analysis indicated a smaller average distance of 0.21 ± 0.01 nm and 0.22 ± 0.01 nm between the WT, Y143R MG ion and drug DTG systems compared to E92Q and G140S each having a distance of 0.41 ± 0.04 nm, 0.99 ± 0.20 nm, respectively. The average number of hydrogen bonds formed between the protein-DNA-MG and drug were calculated to be 1.58, 0.34, 0.20 and 0.54 for the WT, E92Q, G140S and Y143R, respectively (S4A–S4D Fig). The repeats of the simulation runs showed similar results for each of the first simulation runs (S5A and S5B Fig). The RMSD showed equilibrium after 100 ns for the WT system while the G140S mutant system showed increasing RMSD values comparable to the first run (S5A Fig). Additionally, the radius of gyration value was lower for the WT compared to the mutants suggesting the WT structure is more compact compared to the mutant structures, again similar to the first run (S5B Fig). The total non-bonded pairwise interaction energies between the HIV-1C IN protein and DTG were found to be higher for the WT (-94.54 ± 13.20) compared to the three mutant structures (E92Q, Y143R and G140S) each having, -38.74 ± 6.70, -27.97 ± 2.37 and -16.49 ± 1.02 KJ/Mol, respectively. To understand the binding free energy of DTG to the WT and the mutant HIV-1C IN protein structures we performed MMPBSA binding energy calculations. Table 2 summarises the different contributors to the binding free energy. The highest total binding free energy was observed for the WT (-29.65 ± 18.54 Kcal/Mol) followed by the Y143R mutant system (-23.20 ± 10.52 Kcal/Mol) and G140S mutant system (-21.93 ± 23.11 Kcal/Mol), while the E92Q mutant system showed the weakest binding free energy of (-20.65 ± 9.36 Kcal/Mol) (Table 2). The major contributors to the total binding free energy in the WT was the van der Waals energy, electrostatic interaction energy and SASA energy, while the polar solvation had no contribution (because of its positive value) to the binding of the drug and similarly for the E92Q mutant (Table 2). For the Y143R system the van der Waals, polar solvattion and SASA energy were the major contributors to the binding free energy, while the electrostatic interaction energy had no contribution. Both the van der Waals and the electrostatic interaction energies contributed significantly to the total binding energy observed in the G140S mutant, while the polar solvation and SASA energy each had smaller contributions to the binding of the drug. This suggests that all the mutants considered may trigger conformational changes in the active site resulting in significantly weak binding of DTG to HIV-1C IN.

**Table 2:** Binding free energies of DTG to various Protein complexes using MMPBSA method.

Energy (Kcal/Mol)	WT	E92Q	Y143R	G140S
ΔE_vdW_	-43.88 ± 22.54	-23.28 ± 9.08	-21.07 ± 10.13	-14.88 ± 12.22
ΔE_elec_	-1.12 ± 11.27	-2.10 ± 5.07	10.23 ± 6.64	-5.00 ± 9.11
ΔE_psol_	20.90 ± 15.84	8.20 ± 9.01	-8.52 ± 11.33	-0.31 ± 19.84
ΔE_SASA_	-5.55 ± 2.43	-3.47 ± 1.33	-3.84 ± 1.95	-1.73 ± 2.04
ΔG_bind_	-29.65 ± 18.54	-20.65 ± 9.36	-23.20 ± 10.52	-21.93 ± 23.11

ΔE_vdW_: van der Waals energy, ΔE_elec_: Electrostatic interaction energy, ΔE_psol_: polar solvation energy, ΔE_SASA_: Solvent accessible surface area energy, ΔG_bind_: Total binding energy.

### Interaction analysis

We performed interaction analysis for five snapshots (every 50 ns) of each of the simulation systems to determine which residues played a role in the binding of DTG to the protein in the WT and mutant protein structures. For the WT system, interactions were observed between the drug DTG and known active site residues D64, D116 and N148, MG ion and also to DNA nucleotides (Table 3). Similarly, interactions were observed between the drug DTG and known active site residue D64, Y143R, N148, MG ion and DNA nucleotides for the Y143R system (Table 3). Interestingly, no active site residue and MG ionic interactions were formed between DTG and the G140S mutant system, resulting in the dissociation of the drug from the binding pocket over time (S6D Fig). On the other hand, the E92Q system showed interactions with one of the active site residues (D116) but no MG ionic interactions (Table 3). S6A–S6D Fig in Supporting Information File shows the different interactions formed between the drug, MG ion and active site residues for snapshots taken at 100 ns for each simulation system.

**Table 3:** Summary of interaction analysis.

Structure	Cluster	Interactions	
		Hydrogen bonds	Ionic
WT	1 (100 ns)	2 (GUA22^a^, D116)	MG
	2 (150 ns)	3 (THY11^a^, D64, D116)	MG
	3 (200 ns)	2 (GUA22^a^, D116)	MG
	4 (250 ns)	4 (THY11^a^, GUA22^a^, D64, D116)	MG
	5 (300 ns)	2 (THY11^a^, N148)	MG
Y143R	1 (100 ns)	4 (THY11^a^, GUA22^a^, D64, N148)	MG
	2 (150 ns)	4 (GUA22^a^, D64, R143, N148)	MG
	3 (200 ns)	4 (GUA22^a^, D64, R143, N148)	MG
	4 (250 ns)	4 (GUA22^a^, D64, R143, N148)	MG
	5 (300 ns)	5 (THY11^a^; GUA22^a^, GUA22^a^, D64, N148)	MG
E92Q	1 (100 ns)	3 (CYT20^a^, D116, P145)	None
	2 (150 ns)	3 (D116, P145, E152)	None
	3 (200 ns)	3 (CYT20^a^, H21, D116)	None
	4 (250 ns)	4 (CYT20^a^, P142, P145, E152)	None
	5 (300 ns)	3 (CYT20^a^, P145, N148)	None
G140S	1 (100 ns)	3 (GUA22^a^, ADE25^a^, ADE27^a^)	None
	2 (150 ns)	3 (GUA22^a^, ADE25^a^, ADE27^a^)	None
	3 (200 ns)	3 (GUA22^a^, ADE25^a^, ADE27^a^)	None
	4 (250 ns)	3 (THY11^a^, GUA22^a^, ADE27^a^)	None
	5 (300 ns)	3 (THY11^a^, GUA22^a^, ADE27^a^)	None

^a^Interactions with DNA nucleotide residues.

Abbreviations of DNA nucleotides: ADE-Adenine; CYT-Cytosine; GUA-Guanine; THY-Thymine. Abbreviations of amino acids: D-Aspartic Acid; E-Glutamic Acid; H-Histidine N-Asparagine; P-Proline; R-Arginine.

## Discussion

Previous studies by Chen *et al*. and Dewdney *et al*. [19, 21] showed the structural impact of mutations Q148H/R and G140S/A on the flexibility of the HIV-1 IN as a mechanism for RAL resistance. Furthermore, Xue and team [46] found that the cross-resistance mutation E138K/Q148K resulted in a reduction in the chelation ability of oxygen atoms in INSTIs to Mg^2+^ in the active site of the mutated intasomes resulting in a reduced binding affinity of RAL and EVG to the protein. Another simulation study also revealed the binding mode of EVG and RAL to HIV-1 IN and the structural mechanism of drug resistant mutants (G140A and G149A) that affect the 140’s loop region spanning residues 140–149 [23]. However, all of these studies only considered HIV-1B IN and protein models of low sequence identity. In this study, we selected three known mutations E92Q, G140S and Y143R associated with RAL, EVG and DTG resistance to investigate their effect on the protein structure of HIV-1C IN and DTG drug binding. The structural modelling of HIV-1C IN considered a homologous template of high sequence identity, and good overall target sequence coverage, compared to previous homology models that considered templates of low sequence identity. We could therefore accurately reconstruct HIV-1C using the close homolog HIV-1B crystal structure as template to infer accurate drug interactions. Further inspection of the overall structure confirmed accurate prediction of more than 90% of domains within the protein structure, compared to the template HIV-1B structure. The quality analysis provided support for the predicted model based on side chain conformations. Stability predictions showed contrasting results to interaction analysis, whereby amino acid substitutions that resulted in a gain of interactions was predicted to be destabilising. The FoldX changes in energy values were similar to interaction analysis for the three mutant structures under investigation. To fully comprehend the effects of individual mutations we opted to use molecular dynamic (MD) simulations to understand the effect of three known mutations on protein movement and drug interactions. MD analyses have shown to be successful in quantifying small changes in protein structures that can affect overall drug binding [47]. Analysis of the change in trajectory of the mutant systems compared to the wild type suggested less stability and higher fluctuation of the G140S mutant system compared to the WT system. We also confirmed the destabilizing effect of the G140S mutant using principal component analysis which suggested larger randomized concerted movement for the G140S mutant compared to the WT, E92Q and Y143R systems. These findings are contradictory to Chen et al. [19] who performed 150 ns simulation studies of the G140S HIV-1B IN mutant system with NAMD and discovered that the 140’s loop of the single G140S mutant system displayed reduced movements using principal component analysis. Their results showed that the single G140S mutation did not adversely affect drug binding. In our case, the 140’s loop region is stabilized by the G140S mutation and we assume that could reduce drug binding. This is supported by pairwise distance analysis confirming a larger distance between the MG ion and drug DTG for the G140S mutant system compared to the WT and Y143R. Furthermore, the total pairwise non-bonded interaction energy was significantly lower for the G140S mutant compared to the WT, suggesting weaker affinity of the drug DTG for HIV-1C IN in the presence of the mutant. Similarly, the binding free energy calculations also showed higher binding energy between the WT HIV-1C IN and DTG and reduced binding for the E92Q, Y143R and G140S mutant systems. These results are in stark contrast to the study of Chen et al. [19] that showed no difference in binding affinity of RAL to the WT and G140S single mutant. Interestingly, the binding free energy in our study for the WT and DTG (-29.65 ± 18.54) was comparable to that found in the Xue *et al*. [22] study (-30.95 ± 0.10), although having ~1.3 Kcal/Mol energy difference. Further interaction analysis was performed to confirm the hypothesis that the G140S mutation could reduce drug binding by extracting structures at different snapshots of the simulation. Here, we found that the G140S mutation resulted in the drug moving further away from the binding pocket. We also observed weaker interactions for the E92Q mutation but stronger interactions for Y143R mutant based on the average number of hydrogen bonds and the total number of polar contacts between the protein and the drug. The model generated in this study can be used to tease out the effects of novel variants. A few limitations of this study are the use of RAL and EVG mutants and not considering novel RAL or DTG mutations and also simulating single instead of double mutations. However, we have yet to identify double mutants within the South African cohort of HIV-1C infected patients. Another limitation is the exclusion of entropy effects due to the lack of computational resources this might have led to under or overestimation of the binding free energy. However, our total pairwise interaction energies also correlate well with RAL binding energies observed in the Chen et al. [19] study with the WT showing higher pair interaction energy compared to the G140S/Q148H double mutant. Future work will include viral fitness assays to determine the effect of mutants E92Q, Y143R and G140S on the HIV-1C virus replication in the presence of DTG.

## Supporting information

S1 Fig Pairwise amino acid sequence alignment between HIV-1C consensus and HIV-1B (PDBID: 5U1C). The conserved DDE motif residues (D64, D116 and E152) are shown in black boxes.
(TIFF)

S2 Fig Tetrameric 3D structure of HIV-1C Integrase in complex with DNA, MG and drug Dolutegravir. Magnesium^2+^ ions (dirty violet spheres), Dolutegravir (brown), DDE motif residues of the protein represented as navy blue sticks and the DNA as a ladder. Each chain/monomer of the protein is labelled and coloured differently.
(TIFF)

S3 Fig PCA analysis for the first two principal components. (A) Graphical representation of PCA of WT vs E92Q systems plotted over the last 200 ns, (B) Graphical representation of PCA of WT vs G140S systems plotted over the last 200 ns and (C) Graphical representation of PCA of WT vs Y143R systems plotted over the last 200 ns.
(TIFF)

S4 Fig The average number of hydrogen bonds formed between the HIV-1C IN protein-DNA-MG and DTG. A) WT, B) E92Q, C) G140S and D) Y143R.
(TIFF)

S5 Fig Trajectory analysis of the repeat of the four simulation systems. A) RMSD backbone deviation of the four HIV1C IN protein simulations and B) The change in Raduis of gyration values for the backbone atoms of the four HIV1C IN protein simulations.
(TIFF)

S6 Fig Interaction analysis for the four simulation systems. (A) Interactions formed between WT HIV-1C integrase structure and DTG taken at 100 ns. (B) Interactions formed between Y143R HIV-1C integrase structure and DTG taken at 100 ns. (C) Interactions formed between E92Q HIV-1C integrase structure and DTG taken at 100 ns. (D) Interactions formed between G140S HIV-1C integrase structure and DTG taken at 100 ns.
(TIFF)
