# Correction: Molecular dynamic simulations to investigate the structural impact of known drug resistance mutations on HIV-1C Integrase-Dolutegravir binding

**DOI:** 10.1371/journal.pone.0234581

**Published:** 2020-06-05

**Authors:** Rumbidzai Chitongo, Adetayo Emmanuel Obasa, Sello Given Mikasi, Graeme Brendon Jacobs, Ruben Cloete

The following information is missing from the Funding statement: R. Chitongo was supported by the South African Research Chairs Initiative of the Department of Science and Technology and National Research Foundation (NRF) of South Africa, award number UID 64751.
