# Supplementary figures and images for: Molecular dynamic simulations to investigate the structural impact of known drug resistance mutations on HIV-1C Integrase-Dolutegravir binding
Source: PLoS One. 2020 May 7;15(5):e0223464. doi: 10.1371/journal.pone.0223464 (PMC7205217; doi:10.1371/journal.pone.0223464)

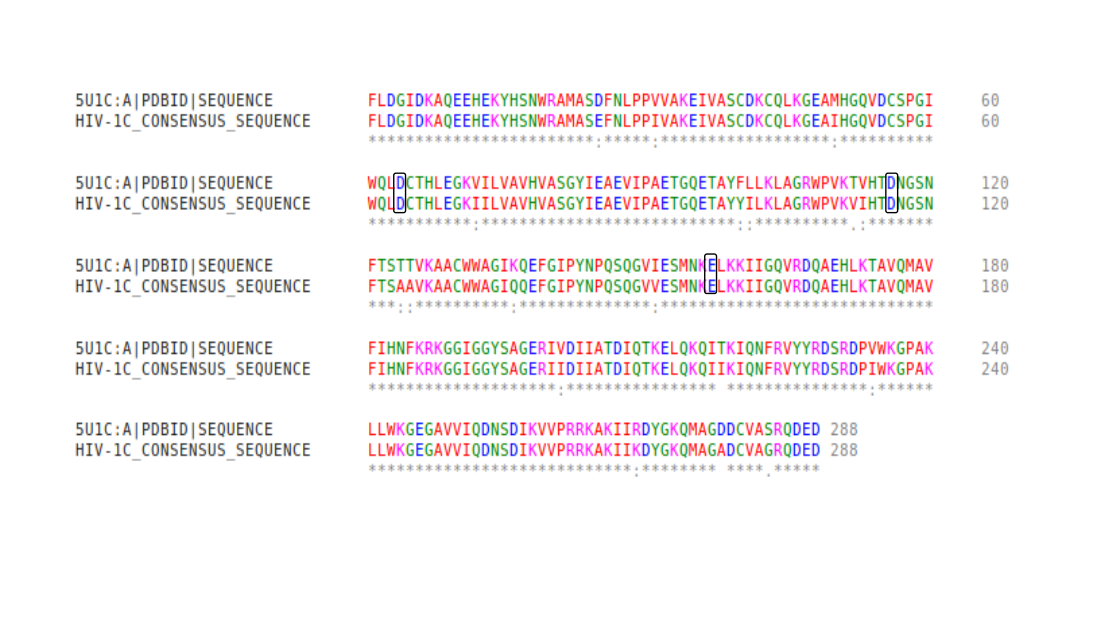

Supplement: S1 Fig — The conserved DDE motif residues (D64, D116 and E152) are shown in black boxes. (TIFF) [file pone.0223464.s001.tiff]

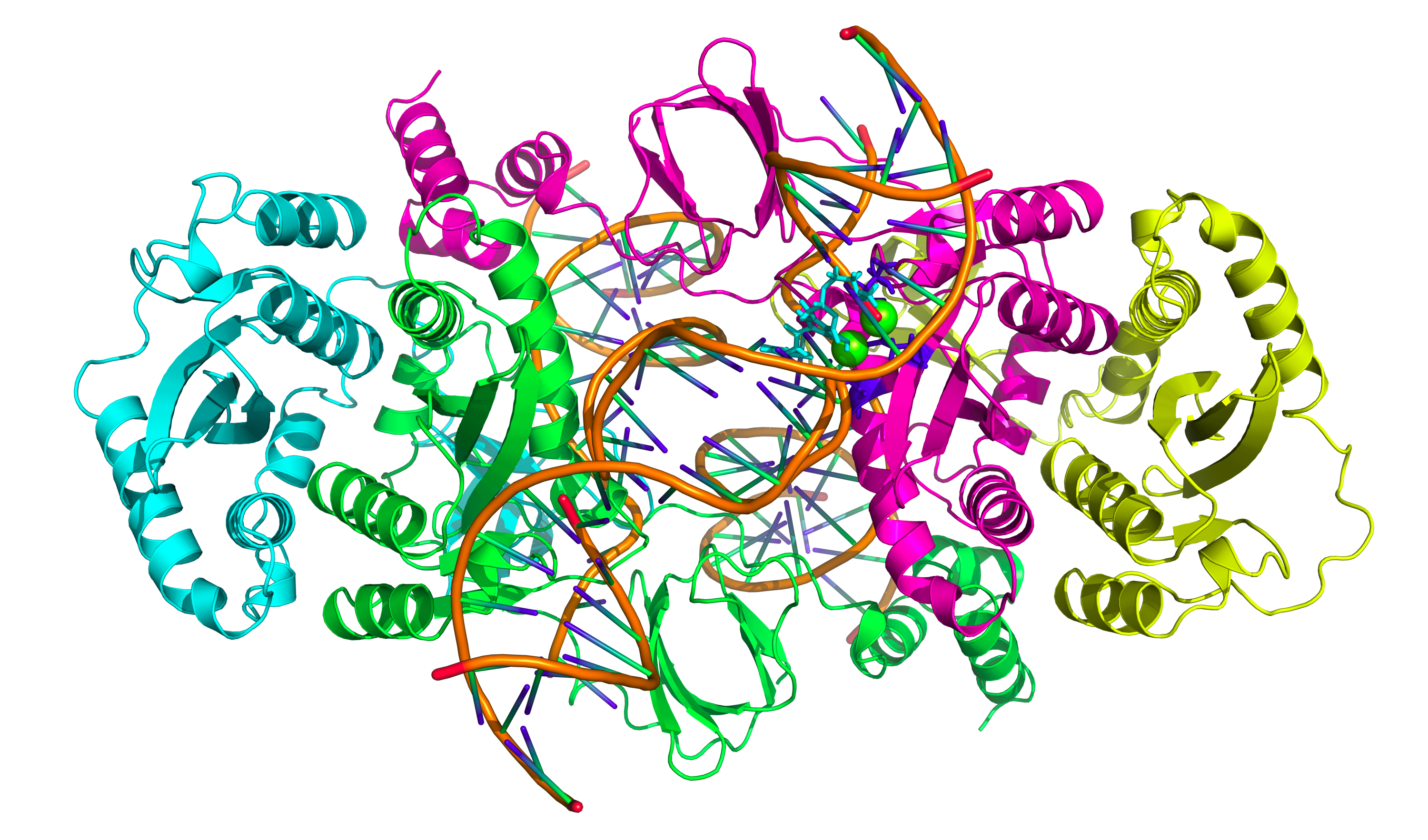

Supplement: S2 Fig — Magnesium2+ ions (dirty violet spheres), Dolutegravir (brown), DDE motif residues of the protein represented as navy blue sticks and the DNA as a ladder. Each chain/monomer of the protein is labelled and coloured differently. (TIFF) [file pone.0223464.s002.tiff]

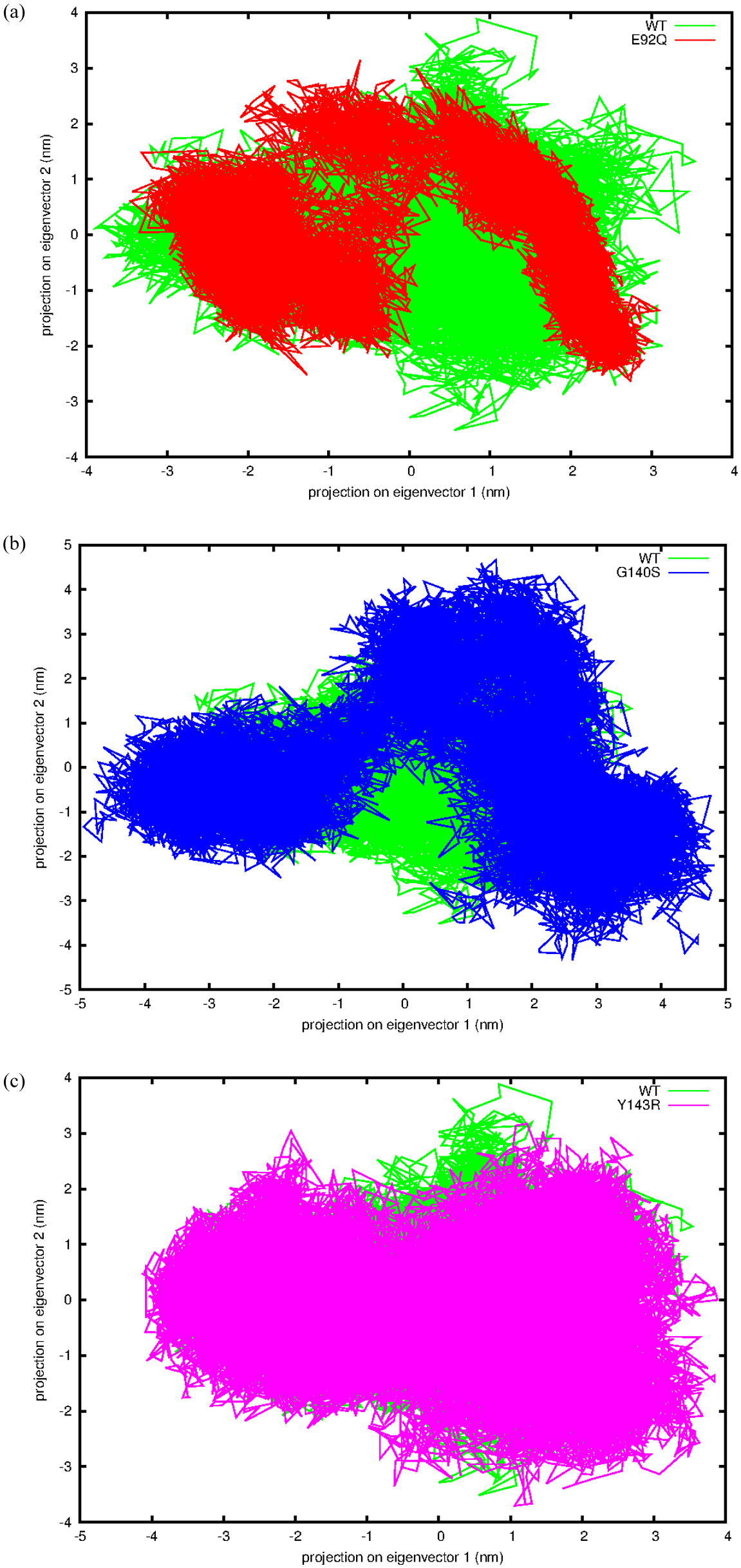

Supplement: S3 Fig — (A) Graphical representation of PCA of WT vs E92Q systems plotted over the last 200 ns, (B) Graphical representation of PCA of WT vs G140S systems plotted over the last 200 ns and (C) Graphical representation of PCA of WT vs Y143R systems plotted over the last 200 ns. (TIFF) [file pone.0223464.s003.tiff]

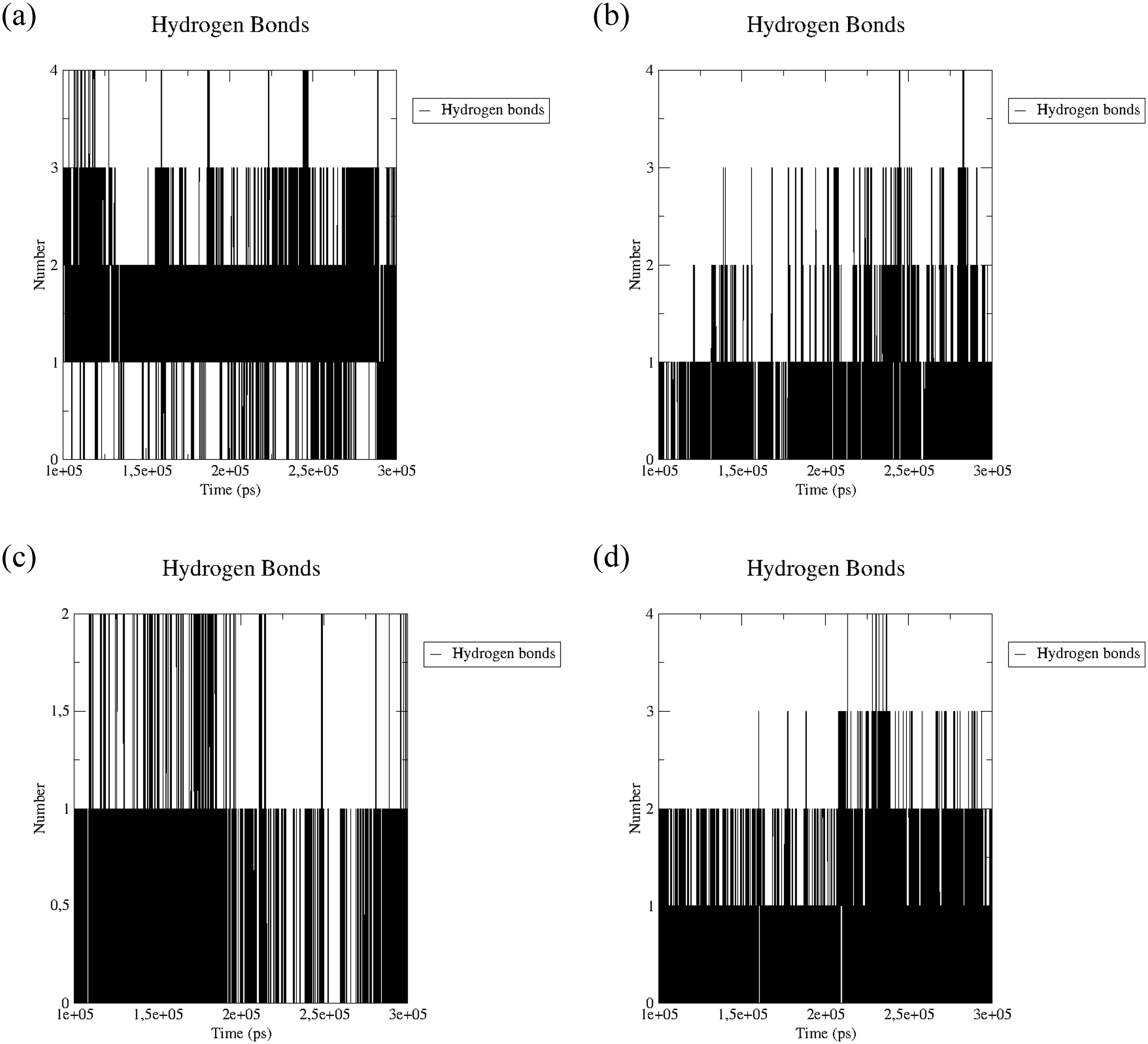

Supplement: S4 Fig — A) WT, B) E92Q, C) G140S and D) Y143R. (TIFF) [file pone.0223464.s004.tiff]

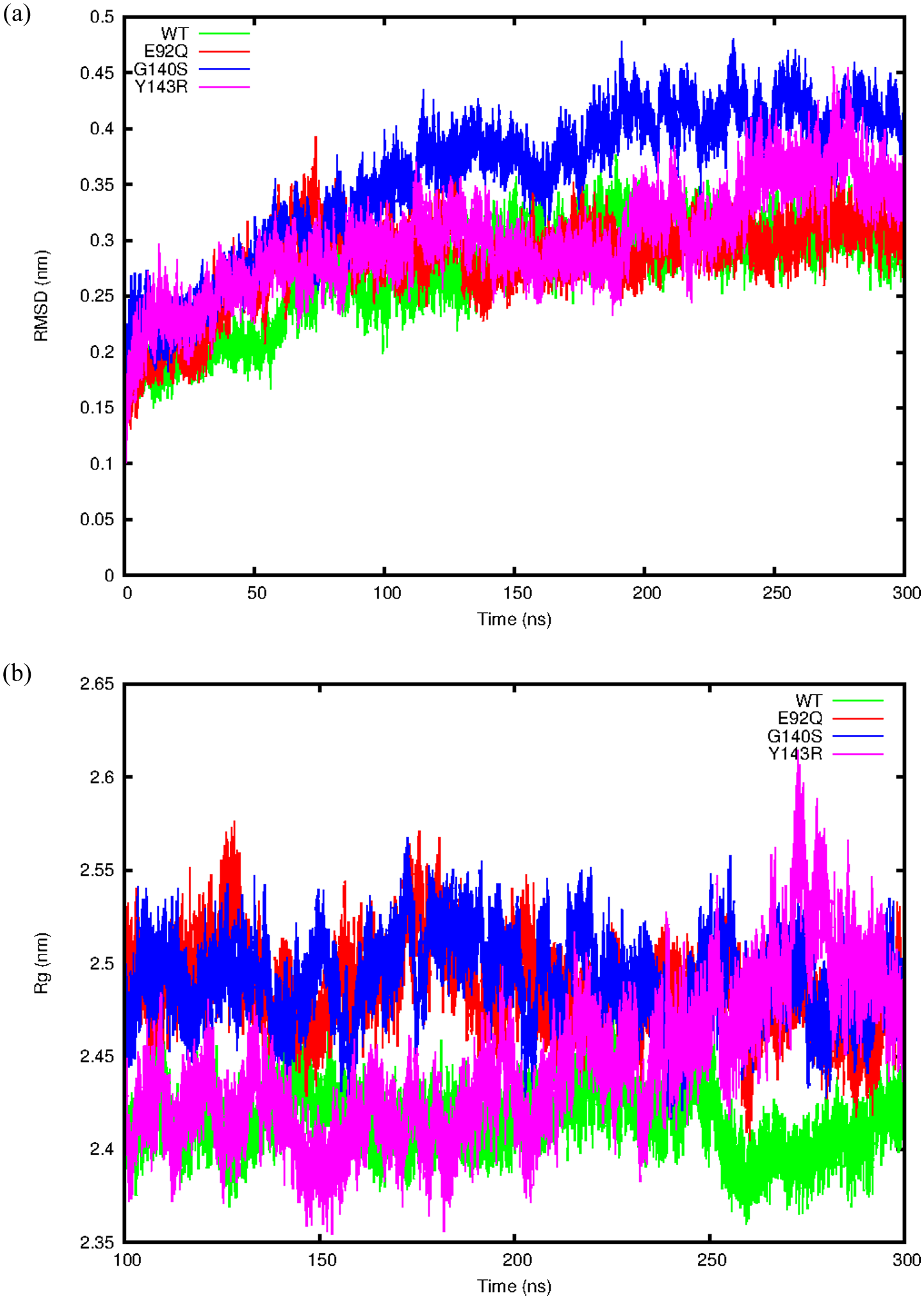

Supplement: S5 Fig — A) RMSD backbone deviation of the four HIV1C IN protein simulations and B) The change in Raduis of gyration values for the backbone atoms of the four HIV1C IN protein simulations. (TIFF) [file pone.0223464.s005.tiff]

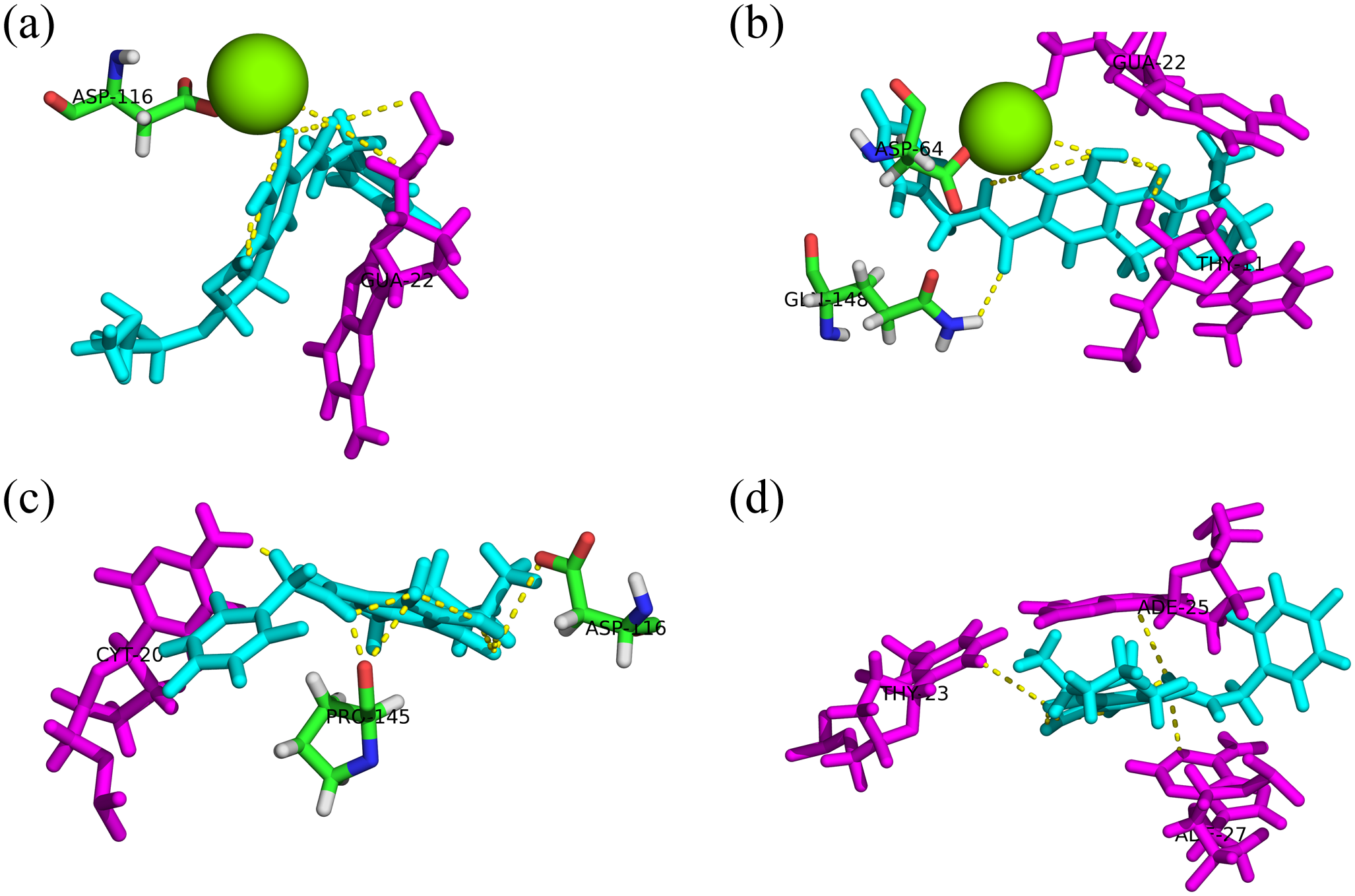

Supplement: S6 Fig — (A) Interactions formed between WT HIV-1C integrase structure and DTG taken at 100 ns. (B) Interactions formed between Y143R HIV-1C integrase structure and DTG taken at 100 ns. (C) Interactions formed between E92Q HIV-1C integrase structure and DTG taken at 100 ns. (D) Interactions formed between G140S HIV-1C integrase structure and DTG taken at 100 ns. (TIFF) [file pone.0223464.s006.tiff]
